# Hypothyroidism in a five-year-old boy with rhabdomyolysis and recent history of cardiac tamponade: a case report

**DOI:** 10.1186/1752-1947-5-515

**Published:** 2011-10-10

**Authors:** Juan José Delgado Hurtado, Waleska Guevara, Evelyn Ramos, Claudia Lorenzana, Susana Soto

**Affiliations:** 1 Universidad Francisco Marroquín, 19 Ave. 8-44 Zona 15, Vista Hermosa I, Guatemala City, Guatemala; 2 Department of Pediatrics, Roosevelt Hospital, 5ta Avenida Calzada Roosevelt, Zona 11, Guatemala City, Guatemala

## Abstract

**Introduction:**

Cardiac tamponade is a rare manifestation of hypothyroidism, and a less rare cause of pericardial effusion. The accumulation of the pericardial fluid is gradual, and often does not compromise cardiac hemodynamic function. There is a relationship between the severity and chronicity of the disease with the presence of pericardial effusion. There are few cases describing associated pericardial tamponade published in the literature. When a tamponade occurs, a concomitant provocative factor such as a viral pericarditis may be related. Our patient's case appears to be the youngest patient described so far.

**Case presentation:**

We report the case of a previously healthy five-year-old Hispanic (non-indigenous) boy who developed rhabdomyolysis with a history of a recent pericardial effusion and tamponade two months before that required the placement of a percutaneous pericardial drainage. Pericardial effusion was considered to be viral. Later on readmission, clinical primary hypothyroidism was diagnosed and thought to be associated with the previous cardiac tamponade. He developed rhabdomyolysis, which was considered to be autoimmune and was treated with steroids. The level of creatine phosphate kinase and creatine kinase MB fraction returned to within the reference rangeone week after our patient was started on steroids and three weeks after he was started on thyroid hormones.

**Conclusions:**

Physicians should consider hypothyroidism as a differential diagnosis in patients with pericardial effusion. Pericardial effusion may progress and cause a cardiac tamponade with hemodynamic instability. The fact that our patient did not have any manifestations of hypothyroidism might have delayed diagnosis.

## Introduction

Pericardial tamponade is a rare complication of hypothyroidism. Previous case series have reported pericardial effusion in 50% to 73% of children with hypothyroidism [1-3] sometimes associated with Down's syndrome [4], but there have been few describing cardiac tamponade associated with hypothyroidism [5]. Aside from bradycardia, cardiac pericardial effusion is the most common cardiac manifestation of clinical hypothyroidism in adults [6]. Effusion in the hypothyroid patient is caused by an increased capillary leak, which increases the albumin rich fluid build-up in the pericardial space and slow lymphatic drainage [7]. Cardiac tamponade is probably rare because the pericardial effusion accumulation is gradual and allows the pericardium to stretch, allowing normal maintenance of cardiac function [8]. Thyroid hormones also have an important effect on the expression of certain gene proteins, including: α myosin heavy chain, potassium channels, β1 adrenergic receptor and a calcium ATPase, which explain the cardiac manifestations. Characteristically, the protein content of pericardial effusion is increased when effusion is due to hypothyroidism. The treatment of pericardial effusion related to hypothyroidism is usually medical. Pericardial effusion generally improves in the hypothyroid patient two months to a year after thyroid hormones are started and the patient becomes euthyroid [4,6,9]. Our patient's case required the placement of a percutaneous drainage due to hemodynamic instability.

## Case presentation

A five-year-old Hispanic (non-indigenous) boy was admitted to our hospital with a history of intermittent fever, and was found to have elevated muscle enzymes.

His mother reported that he had been taken to a doctor five days earlier and the apparent diagnosis was an intestinal infection, for which he was started on trimethoprim sulfamethoxazole and acetaminophen. The fever was associated with productive cough. After no apparent improvement, she decided to bring her child to our emergency department.

**Figure 1:**
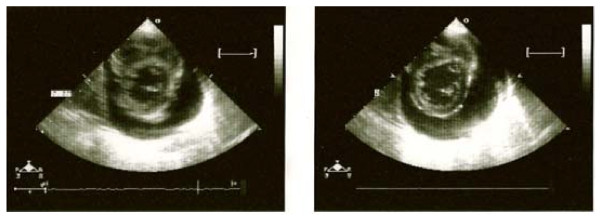
**Echocardiogram showing a large anterior and posterior pericardial effusion causing right atrial and ventricular collapse**.

The child had been healthy four months before. He had been admitted to our hospital two months prior due to dyspnea and hypoxia, and was diagnosed with a pericardial effusion that caused a tamponade on an echocardiogram, causing right atrial and ventricular collapse (Figure 1). Emergency percutaneous pericardial catheter drainage was placed, draining 200 mL of serous clear fluid. A culture from the pericardial effusion was reported sterile; cytology reported no microorganisms or leukocytes, but an increased level of proteins (3400 mg/dL). Acid-fast stain results were negative, as were the results of adenosine deaminase testing. Rheumatoid tests were also performed: anti-double-stranded DNA results were negative and serum complement (C3 and C4) levels were within normal limits. HIV rapid test and serum antibody (IgM) tests for cytomegalovirus and Epstein Barr virus were negative as well. Coxsackie serology was not available. Hypoalbuminemia was also noted at 2 g/dL (reference range 3.4 to 4.8 g/dL) at admission, associated with proteinuria. Tests were inconclusive and the pericardial effusion was considered to be viral. Because of his overall health improvement, he was discharged from hospital one month later, with follow-up planned.

Our patient did not have any important family, traumatic, or surgical history. He had had viral hepatitis three years previously. No allergies or medication taking were reported. He had normal psychomotor development, and vaccinations had been given on schedule.

On physical examination, his vital signs were within normal limits and he was not febrile. His weight was 13.6 kg and his height 98 cm, considered low stature for his age (Z = -3.9). A thyroid palpation revealed no goiter, nodules or pain. Heart sounds were rhythmic and no pericardial rub was heard. An anterior mid-thoracic scar was noted where the percutaneous pericardial catheter had been placed. He had hepatomegaly that was later confirmed on an ultrasound. Results of an anterior chest X-ray were normal.

Hematology tests on admission revealed a total leukocyte count of 6.83 K/UL, hemoglobin level of 12.5 g/dL and a platelet count of 310 K/μL. His blood chemistry results revealed elevated muscles enzymes: lactate dehydrogenase (LDH) of 1117 (reference range 240 to 480 IU/L), a creatine phosphokinase (CPK) of 2874 U/L (reference range 38 to 170 U/L) and creatine kinase MB (CK-MB) of 60 U/L (reference range 0 to 24 U/L). His renal function tests were within normal limits. Hypertrigliceridemia was also noted at 408 mg/dL (reference range 30 to 200 mg/dL). Urine test results showed 500 mg/dL proteinuria and microscopic analysis showed five to eight erythrocytes per field. During the time our patient was hospitalized, his CPK levels were moderately increased (2435 to 4615 U/L). Our patient did not report muscle pain or weakness, and results of a neurological examination were normal. A repeat echocardiogram was performed due to his history, showing normal systolic and diastolic ventricular function with no pericardial effusion.

**Figure 2:**
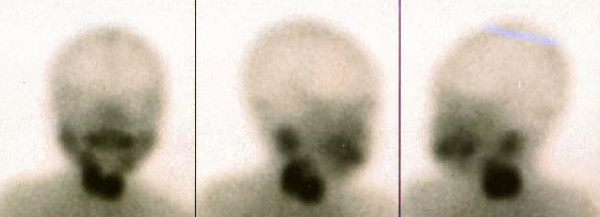
**Thyroid scan with technetium showing a normal absorbing thyroid**.

Considering his recent history of cardiac tamponade, thyroid tests were ordered. The results were diagnostic of primary clinical hypothyroidism: free T3: < 1.1 pg/mL (reference range 1.1 to 3 pg/mL), total T3: 34.9 ng/dL (reference range 100 to 200 ng/dL), free T4: 0.2 ng/dL (reference range 0.7 to 1.85 ng/dL), total T4: 1.04 μg/dL (reference range 4.5 to 12 μg/dL) and thyrotropin 299.380 μIU/mL (reference range 0.490 to 4.670 μIU/mL). A thyroid scan with technetium (Figure 1) and a thyroid ultrasound were performed showing a normally located and absorbing thyroid gland without nodules (Figure 2). Anti-peroxidase (APO) and anti-thyroglobulin antibody test results were negative. Levothyroxine was started at a dose of 9 μg/kg per day. Bone age was also calculated with a radiograph of the left hand and was reported delayed by two years.

**Figure 3:**
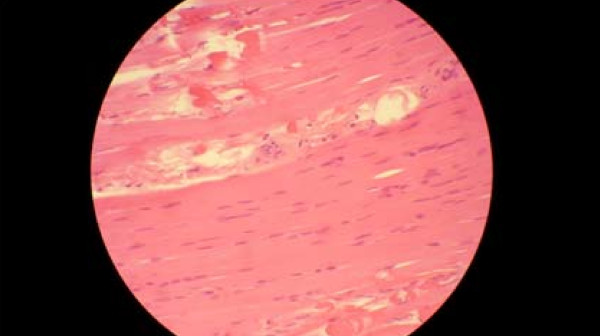
**Muscle biopsy**. Eoshinophilic muscle fibers showing necrosis with no inflammatory cell infiltration.

**Figure 4:**
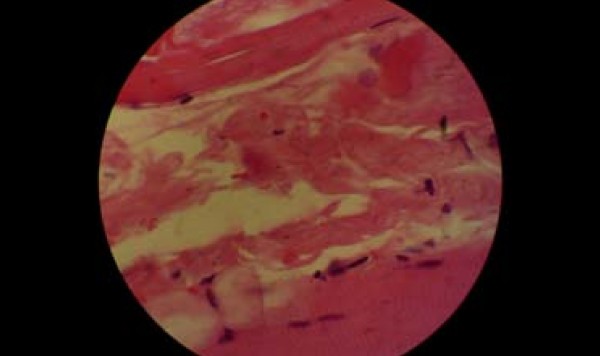
**Muscle fibers with colliquative necrosis**.

An electromyogram reported a myophatic inflammatory process involving mainly proximal muscles compatible with myositis. A muscle biopsy was performed that revealed colliquative necrosis without inflammatory cell infiltration (Figures 3 and 4). An anti-Jo-1 antibody test result was negative. Rhabdomyolisis was, however, considered to be autoimmune and steroids were given. Three weeks after the thyroid hormones and one week after steroids had been started, muscle enzyme levels (CPK, CK-MB, and LDH) had returned to normal levels.

## Discussion

The cause of our patient's hypothyroidism was not diagnosed. Case series and case reports have suggested that there is a relationship between the severity and chronicity of hypothyroidism with the presence of pericardial effusion [2], which can be assessed by the short stature seen in our patient as well as the delayed bone age reported.

Our patient was evaluated by a rheumatologist, who considered that the rhabdomyolisis was probably due to an autoimmune disease. Our patient had moderately elevated muscle enzymes over a period of approximately one month, and there was a great concern acute renal failure might develop. He was given steroids and, fortunately, one week later his muscle enzyme levels had returned to normal. The cause of rhabdomyolysis was not confirmed. Viruses such as Cocksackie can cause pericarditis along with myositis. The fact that the muscle biopsy had no inflammatory cell infiltration might suggest a viral myositis. When a cardiac tamponade occurs in a hypothyroid patient, a concomitant provocative factor can be associated (for example, viral pericarditis) [10]. This could be the triggering infection that exacerbated the cardiac tamponade in a previous hypothyroidism-related pericardial effusion in our patient. Hypothyroidism is a cause of rhabdomyolysis in adults, but has been described in the pediatric population as well [9].

## Conclusions

Cardiac tamponade is a rare manifestation of hypothyroidism. The early diagnosis of pericardial effusion due to hypothyroidism may prevent serious complications such as the cardiac tamponade seen in our patient. As has been described in other case reports, treatment with thyroid hormone replacement reduces the pericardial effusion. The fact that our patient did not have any common clinical manifestations of hypothyroidism might have delayed the diagnosis. Therefore, hypothyroidism should be considered in every patient who presents with a pericardial effusion. Multidisciplinary involvement is important in the treatment of patients with a difficult diagnosis.

## Consent

Written informed consent was obtained from the patient's legal guardian for publication of this case report and any accompanying images. A copy of the written consent is available for review by the Editor-in-Chief of this journal.

## Competing interests

The authors declare that they have no competing interests.

## Authors' contributions

JJD wrote the case report. WG was the doctor in charge of our patient and revised the case report. ER revised the case report. CL and SS were consulting physicians and revised the case report. All authors read and approved the final manuscript.
